# Surgical Surprise and a Ryle’s Tube Misadventure: An Intraoperative Gastric Perforation

**DOI:** 10.7759/cureus.105179

**Published:** 2026-03-13

**Authors:** Srija Koripella, Abhay K Kattepur, Aswathappa D, Shashirekha C A, Sreeramulu P N

**Affiliations:** 1 General Surgery, Sri Devraj Urs Medical College, Kolar, IND; 2 Surgical Oncology, Sri Devraj Urs Medical College, Kolar, IND

**Keywords:** head and neck cancer surgery, iatrogenic gastric perforation, intraoperative complication, nasogastric tube complication, nasogastric tube (ngt), ryle's tube

## Abstract

Nasogastric tube (NGT) insertion is routinely performed for enteral feeding and gastric decompression and is generally considered safe. However, it can lead to complications ranging from minor discomfort, epistaxis, and sinusitis to severe events such as respiratory injury, esophageal perforation, and, rarely, gastrointestinal perforation. Among these, gastric perforation in adults is extremely uncommon.

We describe the case of a 47-year-old woman with carcinoma of the left lateral tongue who underwent near-total glossectomy with bilateral neck dissection, tracheostomy, and reconstruction. After a difficult nasal intubation, a 16F NGT was inserted intraoperatively. During surgery, persistent oliguria and progressive abdominal distention raised suspicion of intra-abdominal pathology. Exploratory laparotomy revealed perforation of the posterior gastric wall caused by the Ryle’s tube. The defect was repaired using a Graham’s omental patch, and the primary procedure was completed. The patient recovered uneventfully.

This case highlights that even routine NGT placement can lead to rare but life-threatening complications. Careful technique, vigilance for atypical intraoperative signs, and reliable confirmation of tube position, preferably through radiographic verification, are essential to prevent and promptly manage such adverse events.

## Introduction

The nasogastric tube (NGT), also known as a Ryle's tube, is commonly used in both acute and chronic settings to provide enteral nutrition and gastric decompression. Although NGT insertion is considered a relatively minimally invasive procedure, it is not without complications. The most common complications include patient discomfort, vomiting, sinusitis, and epistaxis. However, rare complications can be fatal. Injuries and perforations of the alimentary and respiratory tracts are serious but infrequently reported consequences following Ryle's tube insertion.

Mechanical complications after NGT insertion can include respiratory issues, injuries to the esophagus or pharynx, tube blockage, intestinal or intracranial perforations, and accidental tube removal. Additional problems may involve pressure sores from tube fixation and incorrect tube connections [1]. Intracranial perforations tend to occur in trauma patients with skull base fractures; fractures of the cribriform plate can allow the NGT to penetrate into the brain cavity [2,3].

The most serious and frequent complications affect the respiratory system, such as pneumothorax, followed by pleural effusion and aspiration into the lungs, often necessitating chest tube placement. Esophageal perforations happen more often than gastric ones and are more commonly seen in newborns [4]. Gastric perforations in adults after NGT insertion are rare, with only 10 cases documented in the literature [5]. This report details the case of an adult patient who experienced a gastric perforation after intraoperative NGT insertion, presenting during surgery with abdominal distention and decreased urine output.

## Case presentation

A 47-year-old female patient presented with a growth on the tongue. She was evaluated and diagnosed with carcinoma of the left lateral border of the tongue. Initially, she was assessed under anesthesia to determine the extent of the growth and was planned for surgery, followed by adjuvant chemotherapy and radiotherapy.

She was adequately prepared preoperatively and intraoperatively. Following a difficult nasal intubation, a 16F NGT was inserted by the anesthesia team. The planned procedure included near-total glossectomy, marginal mandibulectomy, left-sided modified radical neck dissection, right suprahyoid neck dissection, tracheostomy, and reconstruction with a pectoralis major myocutaneous flap. Intraoperatively, while performing the neck dissection, there was a significant reduction in urine output. Upon confirming the position of the 16F Foley catheter, the procedure was resumed.

Persistently reduced urine output and significant abdominal distention raised concern in the operating room. The pronounced abdominal distention could possibly be due to gastric dilation or hollow viscus perforation caused by the Ryle's tube. After examining the abdomen, diagnostic tapping with a 10 cc syringe was performed. It revealed air in the abdominal cavity, suggesting hollow viscus perforation.

**Figure 1:**
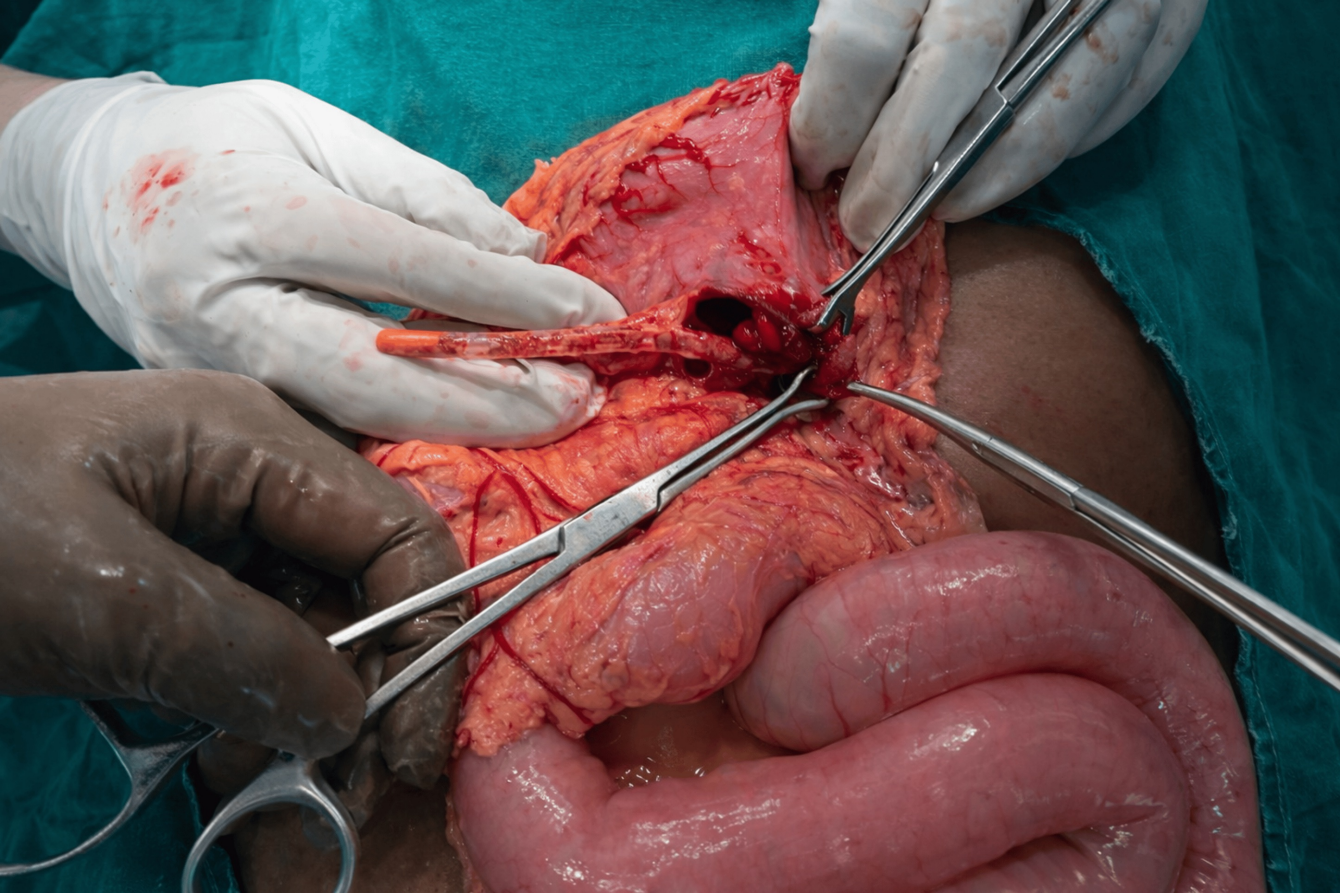
Intraoperative photograph demonstrating a perforation of the posterior wall of the stomach. The presence of blood at the tip of the Ryle's tube confirms a traumatic iatrogenic posterior gastric perforation.

An emergency exploratory laparotomy was performed, revealing the tip of the Ryle's tube penetrating through the posterior wall of the stomach, as shown in Figure 1. The presence of blood at the tip confirmed a traumatic iatrogenic posterior gastric perforation. Due to the abrupt intraoperative nature of the event and technical limitations in the operating room at that time, immediate imaging, such as intraoperative abdominal X-ray or ultrasonography, was not performed. The perforation was repaired using Graham's omental patch, and the remainder of the procedure for carcinoma of the tongue was resumed. The patient was kept nil per oral till postoperative day 5, and the rest of the recovery period was uneventful.

## Discussion

NGT insertion is performed to facilitate fluid administration and gastric decompression. Most serious complications following NGT placement, such as esophageal perforation, pneumothorax, tracheobronchial injury, nasopharyngeal perforation, and even skull base perforation, are iatrogenic [6].

One rare syndrome following NGT insertion is NGT syndrome, which involves paralysis of both vocal cords and supraglottic edema. Although uncommon, this condition can be fatal. The fundamental mechanism involves the tube passing through the muscles near the vocal cords, along with the laryngeal structures being compressed against the spine, which results in an inflammatory response [7].

Acute esophageal necrosis (AEN), also known as black esophagus, increases the risk of esophageal rupture by compromising the integrity of the esophageal mucosa. AEN can result from various causes, including diabetic ketoacidosis (DKA), alcohol use, substance abuse, malnutrition, and critical illness [8-10]. Esophageal candidiasis is another potential infection that can impair esophageal mucosal integrity and lead to esophageal perforation, although it is rare [11].

Possible causes of spontaneous perforation include peptic ulcer disease, gastric malignancy, use of nonsteroidal anti-inflammatory drugs, and gastric ischemia [12].

Adults face a greater risk of perforations in the esophagus and pharyngo-esophageal area after NGT insertion compared to infants, who are more susceptible to gastric perforations [12]. It is still difficult to understand how an NGT can cause a perforation in a healthy, well-vascularized adult stomach. One hypothesis is that pressure ulcers and increased stiffness of the NGT, especially when it remains in place for a long time, contribute to this issue. Tubes left in for over five days have been observed to change color and become stiffer, possibly due to exposure to acidic gastric secretions [13]. Another possible reason is the ongoing irritation from the tube tip pressing against the layers of the digestive tract, a mechanism similar to how ventriculoperitoneal shunts can perforate the gastrointestinal tract [14].

All NGTs must be aspirated and tested to ensure a pH between 1.0 and 5.5. If this pH range is not obtained, a chest radiograph is required to confirm the correct placement of the tube.

While abdominal X-rays are the definitive method for verifying tube placement, other techniques can also be employed. A frequently used alternative involves listening to the stomach with a stethoscope while injecting air through the feeding tube using a syringe. However, this approach is deemed unreliable, and studies have indicated that it may compromise patient safety [15]. 

When conducted and interpreted correctly, an X-ray is the most precise way to differentiate between gastric and lung placement of a newly inserted NGT or nasoenteric tube (NET). It is typically advised for patients at high risk, including those who are critically ill, have impaired consciousness, or lack a gag reflex [1]. The current British National Health Service (NHS) improvement safety guideline recommends using the pH test as the primary method for initial NGT placement verification. This guideline states that a pH of 5.5 or lower is safe and effectively rules out respiratory tract placement [16]. For NETs inserted without imaging guidance, an X-ray remains the most reliable way to confirm the tube tip’s location.

However, delayed migration of a functioning NGT presenting as gastric perforation has also been reported in the literature [17].

## Conclusions

Inserting an NGT is the most natural way to provide enteral nutrition to patients, both acute and chronic, who cannot eat by mouth. However, there have been cases of splenic injuries caused by blindly inserting feeding tubes.

This highlights the importance of healthcare providers to create evidence-based protocols to ensure the correct placement and positioning of feeding tubes. It is important to be aware of factors that may compromise esophageal or gastric mucosal integrity, thereby increasing the risk of perforations during NGT placement. Physicians must carefully assess these risk factors before inserting NGTs to minimize the risk of perforations, a complication associated with high morbidity and mortality.
